# Monitoring and managing microbes in aquaculture – Towards a sustainable industry

**DOI:** 10.1111/1751-7915.12392

**Published:** 2016-07-24

**Authors:** Mikkel Bentzon‐Tilia, Eva C. Sonnenschein, Lone Gram

**Affiliations:** ^1^ Department of Biotechnology and BiomedicineTechnical University of DenmarkMatematiktorvet Bldg. 301DK‐2800Kgs. LyngbyDenmark

## Abstract

Microorganisms are of great importance to aquaculture where they occur naturally, and can be added artificially, fulfilling different roles. They recycle nutrients, degrade organic matter and, occasionally, they infect and kill the fish, their larvae or the live feed. Also, some microorganisms may protect fish and larvae against disease. Hence, monitoring and manipulating the microbial communities in aquaculture environments hold great potential; both in terms of assessing and improving water quality, but also in terms of controlling the development of microbial infections. Using microbial communities to monitor water quality and to efficiently carry out ecosystem services within the aquaculture systems may only be a few years away. Initially, however, we need to thoroughly understand the microbiomes of both healthy and diseased aquaculture systems, and we need to determine how to successfully manipulate and engineer these microbiomes. Similarly, we can reduce the need to apply antibiotics in aquaculture through manipulation of the microbiome, i.e. by the use of probiotic bacteria. Recent studies have demonstrated that fish pathogenic bacteria in live feed can be controlled by probiotics and that mortality of infected fish larvae can be reduced significantly by probiotic bacteria. However, the successful management of the aquaculture microbiota is currently hampered by our lack of knowledge of relevant microbial interactions and the overall ecology of these systems.

Year by year the human population increases in size passing the 7 × 10^9^ mark in 2011, and likely reaching approximately 10^10^ individuals over the course of the coming 30 years (United Nations, Department of Economic and Social Affairs, Population Division, 2015). This growing population is in need of a steady supply of high‐quality protein, which to an increasing degree is being supplied by shell‐ and finfish meat. In terms of greenhouse gas emissions, this trend is commendable as the diet of fish eaters on average emits approximately 50% less greenhouse gases than that of meat eaters (Scarborough *et al*., 2014). However, the increasing need for seafood cannot be met by capture fisheries alone as several fish stocks have been depleted. The most recent estimates suggest that only 9.9% of fish stocks exhibited abundances above their maximum sustainable yield and thus more than 90% of all fisheries are currently maximally exploited, or overexploited (Food and Agriculture Organization of the United Nations (FAO), Fisheries and Aquaculture Department. The State of World Fisheries and Aquaculture (SOFIA), 2014). Hence, it is highly advantageous if the continued increase in production of seafood can be obtained from cultured species rather than capture fisheries. Indeed, the aquaculture industry has contributed with an increasingly large proportion of seafood production since the seventies (Fig. 1), and more than half of the seafood produced in 2013 and 2014 were supplied by the aquaculture industry. However, the industry is faced with a series of challenges on its own.

**Figure 1:**
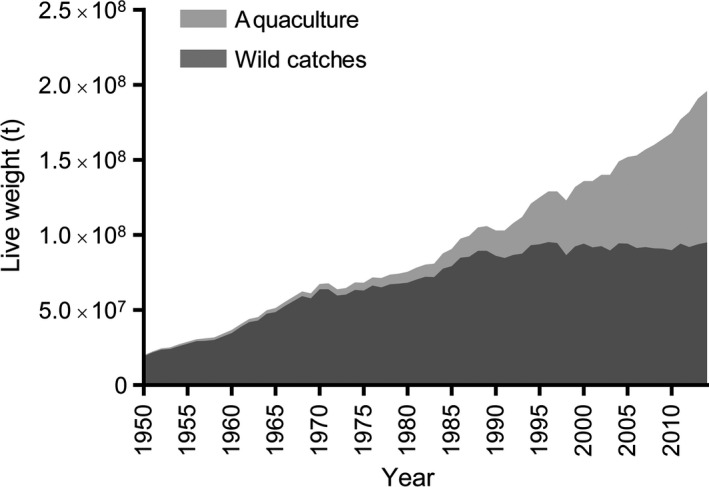
The relative contribution from aquaculture and wild catches to the total increase in seafood production from 1950 to 2014. Data were obtained from the Fisheries Global Information System (FIGIS) at the Fisheries & Aquaculture Department, The Food and Agricultural Organization of the United Nations (FAO).

Fish are usually reared at high densities and at large scale, which pose some challenges with respect to the maintenance of proper water quality. This is seen within the systems, where self‐pollution by inorganic nutrients, fish faeces, and feedstuff remnants poses a problem, but also in the surrounding environment where high‐nutrient effluent may stimulate eutrophication. Other challenges faced by the aquaculture industry relate to the infection of fish or live feed by pathogenic microorganisms. Fish larvae are in particular prone to microbial infections and controlling the growth of these pathogens by conventional means, i.e. by the use of antimicrobials, can pose a severe risk to human health due to the spread of microbial antibiotic resistance (Cabello *et al*., 2013). Nevertheless, microorganisms may also provide solutions to these problems as data suggest that manipulation of the microbial communities associated with the fish and their environment can not only improve water quality in terms of nutrient levels but also reduce the abundance of fish pathogenic bacteria, and improve larval survival, circumventing the need to apply antimicrobials. In the following, we will address some of the challenges faced by the aquaculture industry and how analyses and manipulation of the aquaculture microbiota may improve sustainability and yield.

## Monitoring and improving water quality and animal health in aquaculture using microorganisms

Microbial communities in natural aquatic environments respond rapidly to changes in their immediate environment. These changes may be subtle and may manifest themselves as activation or inactivation of certain metabolic pathways, or they may cause changes to the overall microbial community composition and functionality. With the rapid development of high‐throughput sequencing (HTS) technologies, such as portable, real‐time sequencers, it has become possible to monitor such changes using an all‐encompassing systems biology approach, studying the combined genomic and/or transcriptomic makeup of a single sample. Although not yet standardized, initiatives to incorporate such HTS technology in environmental monitoring programmes are underway for natural marine systems (Bourlat *et al*., 2013; Ininbergs *et al*., 2015), and such approaches hold great potential for aquaculture systems as well. Furthermore, pipelines for the analyses of –omics data and optimization of metabolic processes are being developed by modelling metabolic networks of microbial communities utilized in bioindustries (Zelezniak *et al*., 2015; Perez‐Garcia *et al*., 2016).

Besides the commonly known technical challenges associated with the application of HTS technology to natural samples, e.g. extraction of representative environmental DNA, PCR amplification bias in targeted amplicon sequencing approaches and rapid degeneration of RNA, etc., application of HTS as a tool to monitor the overall state of aquaculture systems is currently hampered by our level of understanding of the microbial ecology of these systems. Hence, the initial prerequisite for the application of HTS technology as a monitoring tool is the characterization of both the healthy and diseased aquaculture microbiome. Studies investigating the microbial community composition in recirculating aquaculture systems (RAS; Schreier *et al*., 2010) and the microbiome of cultured fish have recently been started (e.g. Llewellyn *et al*., 2014), yet the detailed characterization of the aquaculture environment in respect to its residing microbiota and its functions are in its infancy. An additional obstacle is that these aquaculture microbiomes are likely to be system specific and different practices will likely have to be developed depending on the system at hand.

A prerequisite for using microbiome characterization as an indicator of aquaculture system performance is the identification of suitable qualitative microbiological and/or chemical indicators of stress. Suggestions for indicators for the assessment of anthropogenic impacts on marine environments, such as the level of pollution effects on the ecosystem, have been made (Bourlat *et al*., 2013); however, the aquaculture setting is very different from natural marine systems as these are naturally eutrophied. Chemical properties of the rearing water that are adjusted to keep a suitable water quality include salinity, oxygen concentration and pH. These factors are also some of the strongest environmental drivers shaping aquatic microbial communities (Lozupone and Knight, 2007; Herlemann *et al*., 2011; Meron *et al*., 2011; Wright *et al*., 2012; Campbell and Kirchman, 2013; Liu *et al*., 2015) and, hence, suitable indicators could be related to ion transporters, shifts in salinity sensitive pathways, e.g. prevalence of the Embden–Meyerhof pathway over the Entner‐Doudoroff pathway (Dupont *et al*., 2014), changes in the use of terminal electron acceptors and overall changes in community composition and functionality. Changes in microbial nitrogen (N) and phosphorous (P) transport and metabolism may also give some indications of the state of the rearing water as dissolved inorganic N and P represent some of the most important compounds, both in terms of self‐pollution, and in terms of impact on the surrounding environment. Using the aquaculture microbiome as an indicator for the state of the system is, however, only truly valuable if it can act as an early warning system, and hence early indicators of poor water quality needs to be determined empirically.

In addition to the assessment of water quality, a microbiome‐based monitoring system could also serve as an excellent early warning system for detection of unwanted microorganisms such as toxin‐producing algae, and fish‐ and human pathogenic bacteria and viruses. DNA‐based methodology have been developed over the course of the past years using, e.g. multiplex PCR and DNA microarray‐based assays (e.g. Gonzáles *et al*., 2004; Warsen *et al*., 2004; Gescher *et al*., 2008; Lievens *et al*., 2011; Chang *et al*., 2012). The use of such approaches has the advantage that they usually have low detection limits; however, they require that the specific target organisms have been identified. In contrast, a metagenomic approach encompasses all potential pathogens given that a sufficient sequencing depth is applied.

Alternative to the assessment of the aquaculture health status by looking at the microbiome, biosensors could become an interesting monitoring tool (Prindle *et al*., 2012). Through synthetic biology we could potentially generate strains that detect changes in nutrient composition and report back by giving, e.g. a luminescent signal. However, this would include the utilization of genetically modified organisms, which remains a controversial topic in scientific and societal regards.

### Directing microbial communities

With the characterization of the aquaculture microbiome comes also the prospect of directing the microbial community to select for certain community functions that, e.g. improve water quality; either through fertilization of the environment, by which one can select for a favourable microbial community, or by supplying the systems directly with defined microbial assemblages. One, already available, approach to manipulating the microbiota is biofloc technology. The basics of this technology revolve around altering the nutrient limitation of the heterotrophic microbial communities in high‐intensity, zero‐exchange aquaculture systems. Usually, heterotrophs will be carbon (C) limited in these highly N and P loaded systems, and simply, by adding a readily accessible and cheap C source, such as molasses or plant material, the heterotrophic bacteria will be able to take up a substantial fraction of the N present in the systems. In addition to a better water quality, this leads to a significant increase in bacterial biomass, resulting in the formation of bacterial macroaggregates (flocs), serving as a food source for the cultured fish species (Emerencio *et al*., 2013).

Transplantations of microbial communities from different marine systems can alter ecosystem functionality (Reed and Martiny, 2013), and adding a favourable microbiota is to some extent already a common practice when considering ‘green water’ technology in aquaculture. In the farming of non‐filter feeding marine species, specifically in the rearing of their larvae, phytoplankton may be supplied directly to the tanks. Here, the effects of adding phytoplankton are likely not directly related to feeding, as these larvae feed on protozoans and zooplankton, and it remains unclear whether the positive effects are related to, for instance, nutritional value of the live feed, stimulation of the non‐specific immune system of the larvae, production of certain algae exudates or stabilization of water quality. It is also unknown whether these beneficial effects should be attributed to the phytoplankton itself or other associated microorganisms. In Southeast Asian freshwater aquaculture facilities, the ‘green water’ technique is used at a larger scale in the farming of filter feeding finfish and crustaceans. Here, a community composed of primarily phytoplankton, but also an associated prokaryotic biota, as well as protozoans and zooplankton, is added and/or selected for by chemical fertilization, or by the addition of agri‐ and aquacultural waste products. The community primarily acts as feed in these systems, but likely also improves water quality through oxygenation and removal of inorganic nutrients. One recent estimate suggested that ‘green water’ phytoplankton communities are produced at an annual rate of 2.4 × 10^8^ tons per year (Neori, 2011). Despite the extent of its production and application, the composition and function of these communities are largely unknown. Hence, one feature biofloc and ‘green water’ technology have in common is that the specific mechanisms behind their effects, and the organisms responsible, are largely uncharacterized. Although they undoubtedly have beneficial effects on the systems, in which they are applied, these ‘hope for the best’ fertilization approaches (Moriarty, 1997) likely does not meet their full potential. By closing the knowledge gap in the understanding of the microbial ecology of aquaculture systems, we can likely optimize these approaches and thereby increase the autonomy of pond systems, minimizing exchange with the surrounding environment in the process.

### Recirculating aquaculture systems

Another approach, utilizing improved system autonomy by integration of microbial processes, is the closed, RAS. These represent a means to combine high‐intensity rearing with a minimum of water consumption, and with minimal impact on the surrounding environment. Some estimates have suggested that 80–88% of C, 52–95% of N and 85% of P added to aquaculture systems will end up as dissolved chemicals, gas or particulate material in the rearing water (Wu, 1995; Gutierrez‐Wing and Malone, 2006). RAS technology can significantly minimize the impact on the surrounding environment by keeping this water within the system, reducing the effluent waste stream by a factor of 500–1000 (Gutierrez‐Wing and Malone, 2006). The key in this technology is microbial reconditioning of the rearing water, usually following an initial mechanical filtration step removing particulate organic matter, which reduces the proliferation of heterotrophic bacteria in the downstream biological filtering process. In the biofilters, which are large surface area structures hosting microbial biofilms, autotrophic nitrifying bacteria such as *Nitromonas* spp. (ammonium oxidizers) and *Nitrospira* spp. (nitrite oxidizers) remove toxic ammonia (NH_3_), producing nitrate (NO_3_^−^) in the process. One of the challenges in biofilter nitrification is the incomplete transformation of ammonium (NH_4_^+^) resulting in accumulation of the intermediate nitrite (NO_2_^−^), which is toxic to fish compromising ion regulatory, respiratory, cardiovascular, endocrine and excretory processes (Kroupova *et al*., 2005). Multiple environmental factors may influence the nitrification process, but one factor to consider here is also the prerequisite that two functional groups of bacteria need to coordinate their metabolisms to complete the nitrification process as the canonical view of nitrification implies the joint activity of ammonium oxidizers and nitrite oxidizers. In 2006, it was, however, postulated that a slow‐growing organism able to do ‘complete ammonia oxidation’ (commamox) should have a higher fitness than faster growing groups dividing the NH_4_^+^ and NO_2_^−^ oxidation processes among them, especially in a biofilm setting (Costa *et al*., 2006). This prediction was recently shown to be true with the discovery of the commamox bacterium ‘*Candidatus* Nitrospira inopinata’ (Daims *et al*., 2015) as well as two additional *Nitrospira* species (van Kessel *et al*., 2015). Concordant with the postulations of Costa *et al*., commamox genes have been shown to be prevalent in metagenomes from different aquatic biofilm environments such as river beds and sediments, but also from engineered systems such as activated sludge and drinking water treatment plants (Daims *et al*., 2015). Nitrifiers usually found in RAS biofilters are *Nitrosomonas oligotropha*,* Nitrosomonas cryotolerans*,* Nitrosomonas europaea*,* Nitrosomonas cinnybus*,* Nitrosococcus mobilis*,* Nitrospira moscoviensis* and *Nitrospira marina* (Schreier *et al*., 2010 and references herein), but whether commamox bacteria are present in aquaculture systems, or whether supplementation of RAS biofilters with commamox bacteria could facilitate complete oxidation of NH_4_^+^ to NO_3_^−^ remains unknown, and should be investigated in the coming years.

In contrast to NH_4_^+^ and NO_2_^−^, NO_3_^−^ is usually not considered critically toxic in aquaculture systems. Its accumulation in RAS has, however, recently been associated with abnormal swimming and health issues in some fish species (Davidson *et al*., 2011, 2014). Furthermore, it is one of the most important compounds in terms of eutrophication, and hence it too should ideally be removed through denitrification in the biofiltration process, which poses some problems as nitrification is an aerobic process and denitrification is an anaerobic process. Furthermore, to prevent the relatively slow‐growing nitrifying biofilm to be overgrown by heterotrophs, excess organic C is removed prior to the nitrifying process. However, heterotrophic denitrification requires an input of organic C compounds. One solution is to have multiple, or compartmentalized filters carrying out different processes, but this adds to the already high complexity and cost of RAS. Estimates have suggested that the complicated reconditioning and recirculation of water already increases the investment cost per pound of annual production with up to more than four times that of conventional pond systems (Losordo *et al*., 1998). One way to circumvent the need for multiple filters is the potential use of biological aerated filters (BAF), relying on moderate aeration (0.5–3.0 mg O_2_ L^−1^) and the formation of an O_2_ diffusion gradient in the biofilms with denitrifying organisms being situated in the lower, anoxic layers of the biofilm (Puznava *et al*., 2001). Similar to many of the other aspects of RAS, the idea of BAF originates in wastewater treatment. How it performs in the aquaculture setting is, however, something we will have to investigate and evaluate in the years to come.

**Figure 2:**
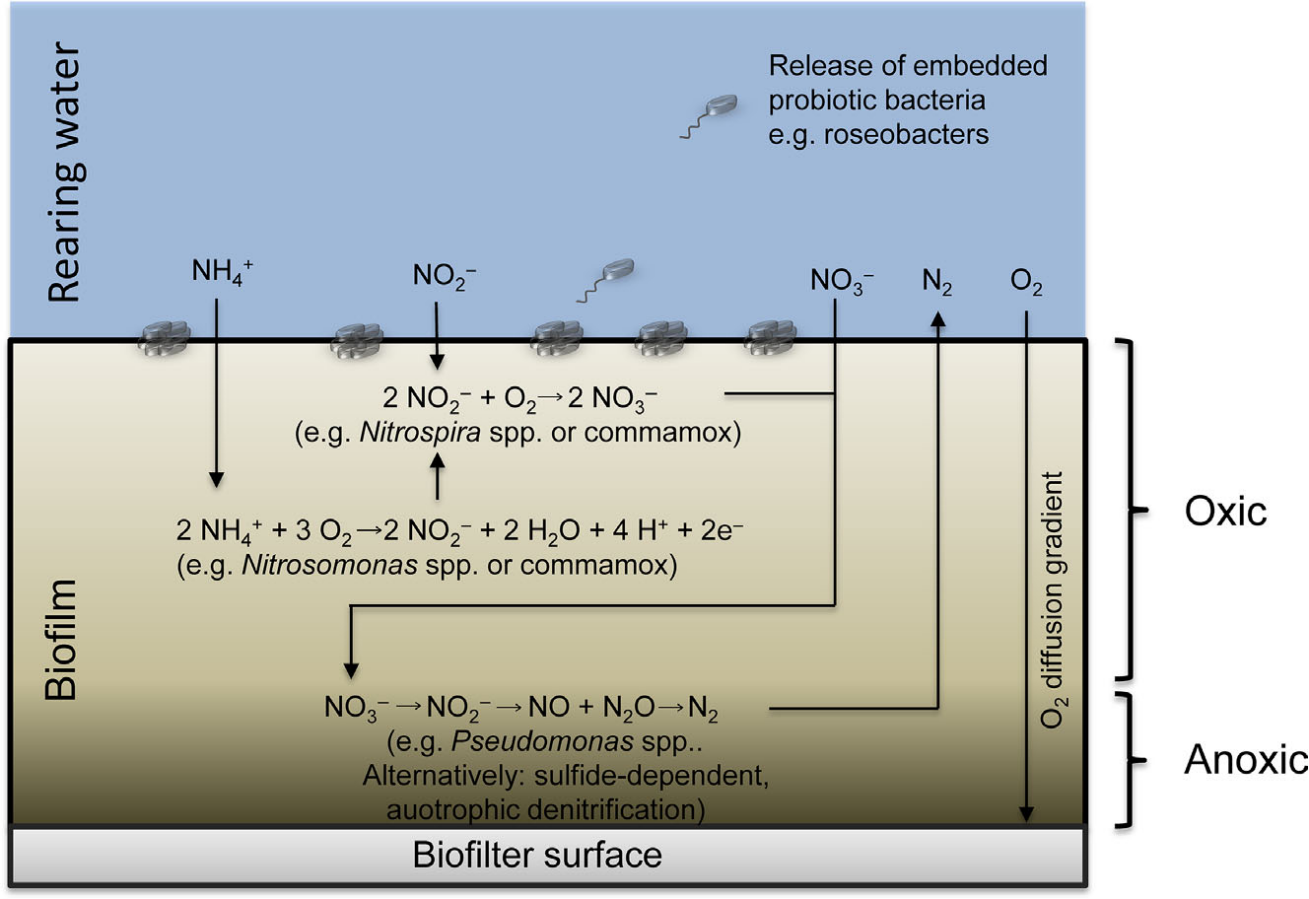
Suggested processes and organisms to incorporate in the design of a synthetic biofilm community in a biological aerated filter (BAF) for use in microbial reconditioning of rearing water. Denitrification is carried out in the anoxic, bottom layer by heterotrophs or autotrophs, whereas nitrification takes place in the upper oxic part of the biofilm. Other processes and organisms could be included, e.g. annamox bacteria or archaea, in the bottom layer, and, potentially, probiotic bacteria could be embedded in the upper layer seeding the rearing water upon release from the biofilm.

One possibility for integrating BAF in RAS and optimizing biofiltration technology is the rational design and engineering of pre‐established biofilms. Currently, biofilters rely on the colonization by largely uncharacterized organisms from the rearing water and subsequent succession and maturation. Particularly, in marine RAS, this process is in itself a bottleneck as it may take up to 3–4 months for a new filter to mature and perform properly (Manthe and Malone, 1987; Gutierrez‐Wing and Malone, 2006). This may be due to insufficient seeding by microbes, especially the nitrite oxidizers seem to be having difficulties establishing themselves in marine biofilters resulting in early accumulation of NO_2_^−^ (Gutierrez‐Wing and Malone, 2006). By constructing synthetic communities and establishing them on filters prior to installation, these filters could potentially perform immediately. Exactly how such a biofilm should be designed would be based on the characterization of natural, functioning biofilter‐associated biofilms from RAS systems and hence the community composition and functionality of such biofilms needs to be investigated further. Ideally, a synthetic community should contain a lower layer of denitrifying organisms, which would be shielded from O_2_ by the other layers of the biofilm (Fig. 2). Candidates could be members of the *Pseudomonas* genus as these are often denitrifiers and prominent biofilm formers. Furthermore, they are known to be able to establish themselves in RAS biofilters (Borges *et al*., 2008; Michaud *et al*., 2009). Alternatively, denitrification could be carried out by autotrophic bacteria through hydrogen sulfide (H_2_S) oxidation and NO_3_^−^ reduction, which in the process would remove toxic H_2_S from the system (Cytryn *et al*., 2005). There should be a nitrifying layer, preferably containing a commamox organism, in the aerobic, upper part of the biofilm (Fig. 2), and additional microbially facilitated biogeochemical processes such as anaerobic ammonia oxidation (annamox) could be integrated based on careful characterization and evaluation of naturally colonized filters. Thus, it is imperative that we start doing in‐depth microbial analyses of RASs and that we find ways of optimizing the technology, making it more cost‐effective and accessible for aquaculturists around the world.

## Microbial control of fish diseases

One major constraint in fish farming is the proliferation of fish pathogenic microorganisms and subsequent disease outbreaks. Outbreaks can have dire economic consequences for individual fish farmers, and in some cases, entire subsectors may be limited by the spread of infectious diseases (Verschuere *et al*., 2000). Bacterial fish pathogens are in general considered the most important infectious microbes in aquaculture (Meyer, 1991), and the industry goes to great lengths to reduce the number of pathogenic bacteria in their facilities. Besides the application of disinfectants and biocides, antimicrobials may be applied to treat infected fish, and unfortunately also sometimes as a prophylactic measure (Cabello, 2006). The implications of general misuse of antibiotics have become increasingly apparent with the development of antibiotic resistance outpacing the discovery and development of new antibiotics, and with the occurrence of bacterial infections completely untreatable with currently available antibiotics. Hence, it is imperative that we, to the extent possible, substitute the use of antibiotics with sustainable preventive measures.

One measure that has been applied with success is the implementation of vaccination programmes. This strategy is an important component of modern finfish aquaculture and vaccines against an array of fish pathogenic bacteria are available for many different farmed fish species (Sommerset *et al*., 2005; Ringø *et al*., 2014). One example of the success of vaccination is the impact of its application in the production of Atlantic salmon and trout in Norway on the overall consumption of antimicrobials for therapeutic use in farmed fish. From 1987 to 1997 the amount of antimicrobials used in the industry dropped from 4.8 × 10^4^ kg to less than 10^3^ kg (NORM/NORM‐VET, 2013). A level it has largely remained at since. Meanwhile, the production of fish has increased from approximately 5 × 10^4^ metric tons in 1987 to approximately 1.4 × 10^6^ metric tons of fish in 2013. The extension and development of vaccination programmes in the coming years are hence highly commendable, however, fish larvae and bivalve molluscs do not have developed adaptive immune systems, and hence vaccination is not applicable in larviculture and the rearing of bivalves. Here, a sustainable alternative approach to the prophylactic administration of antibiotics may be manipulation of the bacterial community associated with the larvae, the rearing systems or in the live feed by e.g. addition of probiotics.

### Probiotic bacteria in aquaculture

One group of probiotic bacteria that have been investigated intensely is lactic acid bacteria, and reports of positive effects using these bacteria as probiotics in larviculture are ample (e.g. Gatesoupe, 1991, 1994; Carnevali *et al*., 2004; Venkat *et al*., 2004). Furthermore, they fulfil some of the, recently expanded (Merrifield *et al*., 2010), criteria for being suited probionts in aquaculture, such as the ability to colonize intestinal mucus and exhibit resistance to low pH and bile salts. These traits imply a focus on the gastrointestinal tract, however, in fish larvae the gastrointestinal tract does not represent an isolated microbiome as such, but the entire larva‐associated microbiota is rather an extension of the microbiota of the surrounding environment. Probionts that exert their probiotic effect in association with gills, skin, feed, water or abiotic surfaces are likely equally, or more relevant in larviculture. It has been suggested that quorum sensing systems of the pathogenic bacteria could be a suitable target for probiotic bacteria in and outside the gastrointestinal tract as the expression of virulence factors in fish pathogens, like e.g. *Aeromonas hydrophila*,* Vibrio anguillarum* and *Vibrio harveyi* is controlled by quorum sensing (Defoirdt *et al*., 2004). Candidate probiotics that potentially could assert their biological control effects in the extended larviculture environment include species of the genus *Bacillus*, which have been shown to reduce the expression of virulence factors in *A. hydrophila* through quorum‐sensing inhibition, increasing the survival of fish in challenge trials as well (Chu *et al*., 2014). Live feed for larvae, such as rotifers, brine shrimp and phytoplankton (live feed for bivalves and for larger live feed) may be important vectors for pathogens and *Bacillus* strains have also been shown to exhibit probiotic effects in this setting, i.e. in brine shrimp cultures (Niu *et al*., 2014).

Members of the *Roseobacter* clade have shown great potential as antagonists of fish pathogens in the marine larviculture environment including live feed cultures (D'Alvise *et al*., 2012; Grotkjær *et al*., 2016a,b) and in larvae of turbot and cod (Hjelm *et al*., 2004; D'Alvise *et al*., 2013). Their probiotic effect does probably not rely on quorum‐sensing inhibition, but rather on the production of one or more antimicrobial compounds including tropodithietic acid (TDA), indigoidine, tryptanthrin and dicyclic peptides (Bentzon‐Tilia and Gram, in press). Furthermore, the roseobacters seem to be indigenous to the larvi‐ and aquaculture setting with numerous strains isolated from these environments (e.g. Hjelm *et al*., 2004; Grotkjær *et al*., 2016a). This suggests that the roseobacters can establish themselves in the systems, and that they do not have a negative impact on the fish larvae directly. One of the challenges in the implementation of these probiotics is, however, to assess the impact of the probionts on the system as such, which remains unknown. The mode of action of TDA was recently shown to be disruption of the proton motive force (Wilson *et al*., 2016), explaining earlier reports, which noted that TDA is effective against a broad range of Gram‐positive and Gram‐negative bacteria (Porsby *et al*., 2011). Whether the application of TDA‐producing roseobacters can have undesirable effects on the aquaculture microbiota will have to be investigated in comparative studies of microbial community composition and function. An additional concern is how fish pathogens will interact and evolve with added probionts over time. We recently found that continuous exposure of the fish pathogen *V. anguillarum* to sublethal concentrations of TDA did not result in development of resistance (Rasmussen *et al*., 2016), which is in line with previous findings (Porsby *et al*., 2011), nor did it affect virulence in this pathogen. Although TDA‐producing roseobacters have so far not been found to effect algal cultures negatively during co‐cultivation (D'Alvise *et al*., 2012; Segev *et al*., 2016), a few strains are capable of producing algaecidal substances, so‐called roseobacticides (Seyedsayamdost *et al*., 2011). The pure compounds are deadly to some algal strains, however, leave others unharmed. To prevent the transformation from probionts into pathogens that affect any level of the aquaculturing process, they need to undergo extensive virulence screening as is common practice for other areas such as the dairy industry. To do so, routine bioassays on the different organism groups utilized in aquaculture need to be put into place; however, a prerequisite for effective screening using genomic data is the identification of the genes and pathways responsible for pathogenicity. In case potential harmful features are detected in a promising probiont, one option is genetic modification of the strain to delete undesirable traits. As the applicability of genetically modified organisms outside the laboratory is limited, screening of phylogenetically related strains could prove useful. In the case of the roseobacticides, we have indeed identified a strain that shares the probiotic capabilities and does not produce the algaecidal compounds (E. C. Sonnenschein, C. Phippen, M Bentzon‐Tilia, S. A. Rasmussen, K. F. Nielsen, L. Gram, unpublished).

Another challenge is how to introduce probiotics into, e.g. larval rearing facilities, and whether the probionts will work efficiently within such complex systems. Roseobacters are indigenous to the aquaculture environment (Bentzon‐Tilia and Gram, in press, and references herein), and should hence possess the overall abilities to remain in the systems. Steps towards addressing the efficacy of *Roseobacter* probiotics in live feed systems in the presence of aquaculture‐relevant microbial communities have been taken (Grotkjær *et al*., 2016b), yet whether the probionts added to live feed will remain and proliferate downstream in the system remain unknown. By tracing the probionts in different up‐scaling scenarios, this question can be addressed and such studies would provide valuable data for developing and optimizing administration procedures. Continuous additions at different sites in the rearing facility may be an appropriate approach to ensure the continuous presence of probionts, but whether probionts should be supplied as a suspension, freeze‐ or spray‐dried powder, or embedded in, e.g. alginate microcapsules, providing the probionts with essential nutrients or precursors (e.g. iron for TDA‐production in roseobacters; D'Alvise *et al*., 2016) remains to be investigated. In RASs one possibility is the integration of probiotics into the synthetic communities of pre‐established biofilms on BAFs. As the biofilm grows, release of embedded cells could enable the successful spread of probiotics throughout the system (Fig. 2).

Thus, as the aquaculture industry continues to expand we need to develop sophisticated methods for monitoring different aquaculture systems, and for improving their autonomy, reducing the effects that intensive fish rearing has on its surroundings and on human health. To succeed in this, we need to do in‐depth investigations on how the microbial ecology of aquaculture systems works, and we need to identify key processes and key interactions between relevant organisms in these settings over the coming years.

## Conflict of interest

None declared.
